# Dosimetric effect of respiratory motion on planned dose in whole-breast volumetric modulated arc therapy using moderate and ultra-hypofractionation

**DOI:** 10.1186/s13014-022-02014-5

**Published:** 2022-03-05

**Authors:** Mikko Mankinen, Tuomas Virén, Jan Seppälä, Heikki Hakkarainen, Tuomas Koivumäki

**Affiliations:** 1 Department of Physics, University of Jyväskylä (JYU), P.O. Box 35, FI-40014 Jyväskylä, Finland; 2 Department of Medical Physics, Central Finland Health Care District, Hoitajantie 3, FI-40620 Jyväskylä, Finland; 3 Center of Oncology, Kuopio University Hospital (KUH), Kuopio, Finland

**Keywords:** Breast cancer, Whole-breast irradiation, Volumetric modulated arc therapy, Respiratory motion, Ultra-hypofractionation

## Abstract

**Background and purpose:**

The interplay effect of respiratory motion on the planned dose in free-breathing right-sided whole-breast irradiation (WBI) were studied by simulating hypofractionated VMAT treatment courses.

**Materials and methods:**

Ten patients with phase-triggered 4D-CT images were included in the study. VMAT plans targeting the right breast were created retrospectively with moderately hypofractionated (40.05 Gy in 15 fractions of 2.67 Gy) and ultra-hypofractionated (26 Gy 5 fractions of 5.2 Gy) schemes. 3D-CRT plans were generated as a reference. All plans were divided into respiratory phase-specific plans and calculated in the corresponding phase images. Fraction-specific dose was formed by deforming and summing the phase-specific doses in the planning image for each fraction. The fraction-specific dose distributions were deformed and superimposed onto the planning image, forming the course-specific respiratory motion perturbed dose distribution. Planned and respiratory motion perturbed doses were compared and changes due to respiratory motion and choice of fractionation were evaluated.

**Results:**

The respiratory motion perturbed PTV coverage (V95%) decreased by 1.7% and the homogeneity index increased by 0.02 for VMAT techniques, compared to the planned values. Highest decrease in CTV coverage was 0.7%. The largest dose differences were located in the areas of steep dose gradients parallel to respiratory motion. The largest difference in DVH parameters between fractionation schemes was 0.4% of the prescribed dose. Clinically relevant changes to the doses of organs at risk were not observed. One patient was excluded from the analysis due to large respiratory amplitude.

**Conclusion:**

Respiratory motion of less than 5 mm in magnitude did not result in clinically significant changes in the planned free-breathing WBI dose. The 5 mm margins were sufficient to account for the respiratory motion in terms of CTV dose homogeneity and coverage for VMAT techniques. Steep dose gradients near the PTV edges might decrease the CTV coverage. No clinical significance was found due to the choice of fractionation.

**Supplementary Information:**

The online version contains supplementary material available at 10.1186/s13014-022-02014-5.

## Introduction

Right-sided breast cancer has traditionally been treated under free-breathing (FB) conditions using tangential fields. Hypofractionation has shortened the breast cancer treatment courses from 25 to 15 fractions [1] and 5 fractions [2], henceforth called moderate hypofractionation and ultra-hypofractionation. Ultra-hypofractionation is gradually gaining acceptance [3, 4], and will reduce the clinical load and costs in breast cancer treatment [4, 5]. However, the new emerging fractionation schemes have increased the plan quality requirements which are not always achievable with conventional treatment techniques. The volumetric modulated arc therapy (VMAT) technique has been utilized in treating breast cancer, as it has been proven to yield high coverage and improved homogeneity on the target dose [6–8]. While a recent study found VMAT dosimetrically feasible for ultra-hypofractionated left-sided early breast cancer treatments [9], possible dosimetric errors caused by respiratory motion on highly modulated fields have raised a concern.

While the dose deviation caused by respiratory motion has been shown to average out after five fractions in lung cancer phantom [10], the dose-averaging effect in whole-breast irradiation (WBI) remains a question as the breast targets are usually large and the chest wall region may undergo shape changes with expiration and inspiration. Clinically, it is recommended to use respiratory gating if respiratory motion range exceeds 5 mm in any direction [11].

The effects of respiratory motion have been studied for tangential breast cancer treatment techniques [12–16], but the feasibility of VMAT techniques has not been investigated for WBI under free-breathing conditions. Previously, the effects of breathing motion on WBI dose distribution have been simulated using isocenter shifts and weighted end-expiration and end-inspiration dose calculations [12–14]. However, incorporating a realistic respiratory cycle is challenging and few studies using a four-dimensional computed tomography (4D-CT) image set and IMRT technique to investigate the effect of breathing motion on breast irradiation have been published [15, 16]. Another IMRT study suggested that the areas of homogeneous target dose would be unaffected and biggest deviations would be observed close to the target edges [17]. However, no direct conclusion can be drawn for the VMAT technique, where the dose is delivered from a multitude of angles.

This study is the first to evaluate the feasibility of VMAT technique in right-sided WBI under free-breathing conditions by simulating the delivered dose on 4D-CT image sets. In addition, the differences in dose-averaging effects between moderate hypofractionation and ultra-hypofractionation are evaluated.

## Materials and methods

Ten patients originally diagnosed with lung cancer with 4D-CT images were included in this study. The whole-breast target volumes were delineated according to the ESTRO guideline [18]. As acceptance criteria, a real-time position management (RPM, Varian Medical Systems, Palo Alto, CA) dataset and fully imaged breast region with at least 2 cm margins both cranially and caudally were required. The study protocol was approved by Central Finland Health Care District.

Ten phase-triggered 4D-CT images per patient were acquired using Siemens mCT (Siemens Healthcare GmbH, Erlangen, Germany) with 2 mm slice thickness. Patients were instructed to breathe calmly and were imaged in a supine position. End-inspiration and end-expiration phase markers were placed automatically in the RPM data and corrected by a radiotherapist if necessary.

The treatment planning was conducted in the end-inspiration anatomy to simulate a worst-case scenario. Clinical target volumes (CTV) were delineated on the end-inspiration images by an oncologist and expanded by 5 mm to form the planning target volumes (PTV). The PTV and CTV structures were cropped 5 mm inside from the skin to form PTVin and CTVin. In addition, the PTV was expanded 8 mm outside the body to be used in conjunction with a virtual bolus [19]. All target delineations were carried out in Eclipse (version 15.6, Varian Medical Systems) treatment planning system (TPS).

The organs-at-risk (OAR) were automatically delineated on the end-inspiration image using MIM Maestro software (MIM Software Inc, Cleveland, OH) based on national atlas for breast cancer [20] and verified by the planning physicist. The delineated OAR structures were lungs, heart, contralateral breast and liver.

A representative respiratory cycle was formed for each patient by averaging the RPM respiratory cycles. The representative cycles were sampled to the median prefiltered respiratory cycle length for each patient. In addition, the range of chest wall movement perpendicular to the planned tangential field central axis was determined from the 4D series for each patient. The midpoint slice of CTV, in cranial-caudal direction, was chosen as the measurement location. In addition, the liver motion amplitude was measured from the 4D series by measuring the cranio-caudal displacement of the liver dome.

Eclipse (Varian Medical Systems) treatment planning system (TPS) and Monaco TPS (Elekta AB, Stockholm, Sweden) were used in generating the treatment plans. The plans were generated for Varian TrueBeam linear accelerator with Millenium 120 MLC and Elekta Infinity linear accelerator with Agility MLC, respectively. Eclipse used the Photon Optimizer planning algorithm and analytical anisotropic algorithm (AAA) 15.6 for dose calculation while Monaco used the X-ray Voxel Monte Carlo algorithm for dose calculation.

Six treatment courses were planned for each patient using three techniques and two fractionations. Two VMAT techniques, Varian Rapid Arc (RA) and Elekta VMAT (E-VMAT), were used and tangential three-dimensional conformal radiation therapy (3D-CRT) plans with tangential main fields and 2–3 subfields were also generated for reference. The RA and 3D-CRT plans were generated in Eclipse and E-VMAT plans were generated in Monaco. These plans are referred to as original plans in this article. The treatment planning was carried out using moderate hypofractionation (40.05 Gy in 15 fractions of 2.67 Gy). The ultra-hypofractionated plans (26 Gy in 5 fractions of 5.2 Gy) were formed by adjusting the fractionation of the original 15 fraction plans for each technique. No reoptimizing was performed to avoid confounding effects of different planning objectives and differences in dose distributions. The maximum leaf speeds were 25 mm/s and 65 mm/s for RA and E-VMAT, respectively, and the dose rate was limited to 600 MU/min for all plans. The prescribed dose was normalized to the mean dose of PTVin.

An 11 mm virtual bolus was utilized in RA and E-VMAT treatment planning. The VMAT fields were restricted to tangential directions, to better spare the contralateral normal tissue compared to conventional VMAT [8]. Posterior arcs ranged between 181° and 260°–275° and the anterior arcs between 325°–360° and 60°–70° according to individual patient anatomy. The collimator angles for posterior and anterior arc fields were ± 5°–20° for RA and ± 2° E-VMAT. The planning goal was that 95% of the prescribed dose covered 95% of the PTVin and 98% of the CTVin. The V107% was limited to 1 cc. The mean dose of ipsilateral lung was limited to 20% of the prescribed dose. In addition, the volume of 16 Gy dose was limited to 20% of the ipsilateral lung. Similarly, mean doses to contralateral lung, breast and heart were limited to 1 Gy. In addition, the normal tissue V110% was limited to 1 cc.

**Fig. 1:**
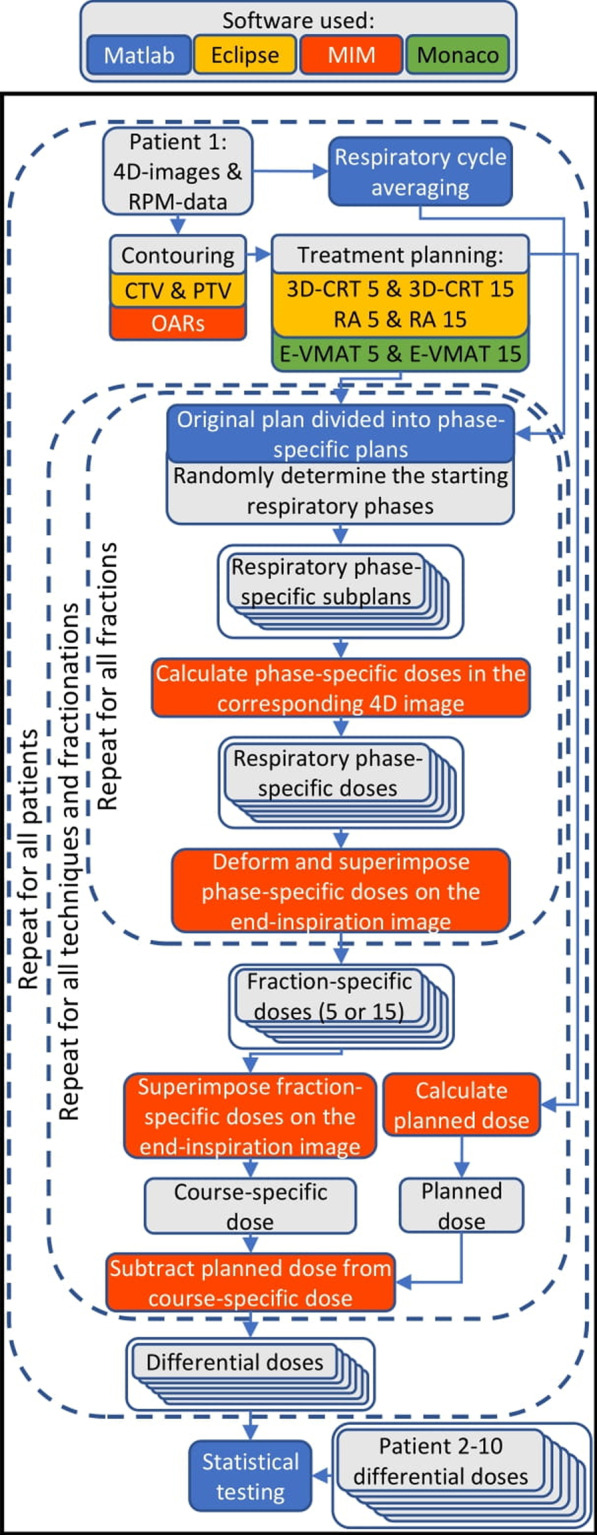
Flowchart representing the data processing. Loop structures are indicated by dashed rounded rectangles. General information and data are represented by gray background while other colors indicate the software used

A workflow was designed to simulate the respiratory motion perturbed dose for each treatment course using the original Eclipse and Monaco plans and the RPM data acquired during patient imaging (Fig. 1). SureCalc Monte Carlo dose calculation algorithm was used to calculate all dose distributions in MIM Maestro software with generic beam models for Varian TrueBeam and Elekta Infinity linear accelerators. In this article, the planned dose refers to the dose distribution calculated in MIM. The initial dose distributions calculated in Eclipse or Monaco were not used in the analysis.

**Fig. 2:**
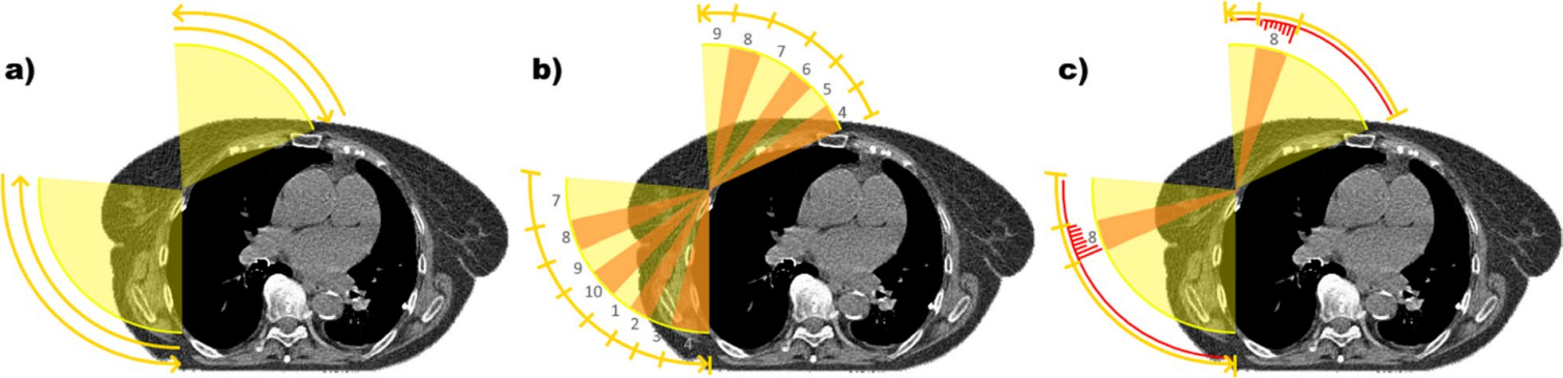
The original VMAT plan with four arcs **a** is divided into respiratory phase-specific subplans (**b**). The starting respiratory phases were randomly determined for the first anterior and posterior arcs (phases 4 and 7, as an example). Image on the right (**c**) demonstrates these arcs in a single phase-specific subplan that only contains the irradiation coinciding with, for example, the eighth respiratory phase. The MUs delivered in this phase-specific subplan are indicated with bars. Dose rate is zeroed between CPs not coinciding with the eighth respiratory phase

A custom-made Matlab script (2020b, MathWorks Inc, MA, USA) was used to divide the original plans into respiratory phase-specific subplans, that is a part of the original plan that would be irradiated during a given respiratory phase. In the phase-specific plans, the dose rate was zeroed between control points (CP) not coinciding with the respiratory phase. For VMAT plans, the starting respiratory phases for the first anterior and posterior arcs were randomly determined for each fraction (Fig. 2). The starting phases of the second anterior and posterior fields were subsequent to the last phase of the preceding arc. The gantry angles coinciding with the respiratory phases were solved using the representative respiratory cycle and gantry rotation speed. CPs were added to the plans with a tolerance of ± 0.1°, if the respiratory phase changed between the original CPs.

Similarly, the starting respiratory phases of anterior and posterior 3D-CRT fields were determined randomly, and the plans were divided into phase-specific subplans, according to the amount of monitor units (MU) per field. Average pauses between fields, 2.1 s after the open field and 1.1 s between subfields, were adapted into the division algorithm.

The phase-specific dose distribution of each phase-specific plan was calculated in the corresponding respiratory phase image. The phase-specific dose was then deformed and superimposed onto the end-inspiration phase planning image. The sum of all deformed phase-specific distributions represented the dose delivered in one treatment fraction. The process of dividing the original plans into phase-specific plans was then repeated for all fractions included in the original plan (total 5 or 15 times). The random starting respiratory phases for the first anterior and posterior arcs or fields were resampled for each fraction.

Once all the fraction-specific dose distributions were calculated for a given technique and fractionation, they were summed to form the final respiratory motion perturbed course-specific dose distribution. Thus, a total of 6 course-specific dose distributions were simulated per patient. Finally, the planned doses were calculated according to the original plans in MIM, and differential dose distributions were formed by subtracting the planned doses from the corresponding course-specific doses.

Dose volume histograms (DVH) were compared between the planned and respiratory motion perturbed distributions for both fractionations. In addition to the planning objectives, the maximum dose to 1 cc volume (D1cc) was evaluated for all structures. The minimum dose to 1 cc (Min%), conformity index (CI) and homogeneity index (HI) were evaluated for PTVin and CTVin. HI and CI were calculated using formulas (D2%–D98%)/D_prescription_ and V95%_total_/V_structure_, respectively. The statistical difference between the DVH parameters was determined by Wilcoxon signed rank test (*p* < 0.05).

The differential distributions were deformed to an anatomy of one patient to localize the statistically significant differences between planned and respiratory motion perturbed dose. Student's *t* test was performed on the differential distributions on a voxel-by-voxel basis including adjacent neighboring voxels. The significance level was adjusted using Benjamini–Hochberg method [21]. Furthermore, an average of the differential distributions was formed in the chosen patient anatomy.

## Results

**Fig. 3:**
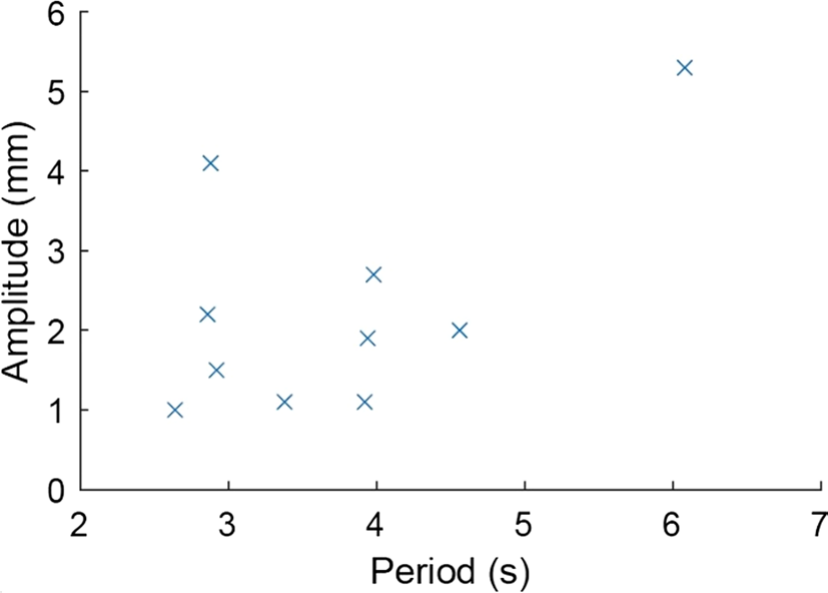
The respiratory periods versus chest wall amplitudes across all patients

**Fig. 4:**
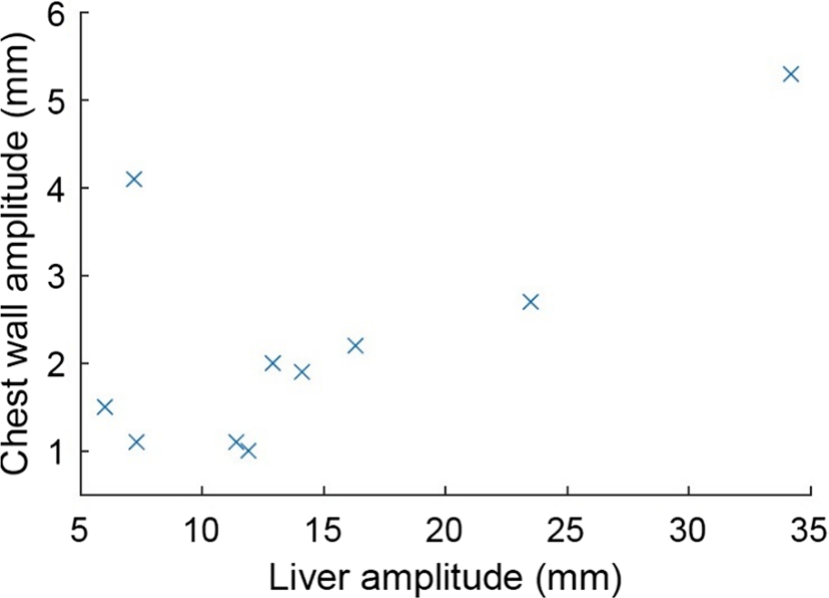
The cranio-caudal liver and the chest wall amplitudes with respiratory motion across all patients

The respiratory motion characteristics across all patients are presented in Figs. 3 and 4. The range of chest wall movement exceeded 5 mm for one patient. This patient was excluded from the further analysis and reported separately, as respiratory gating is recommended for respiratory motion larger than 5 mm [11]. The average range of chest wall movement between end-inspiration and end-expiration phases, perpendicular to the 3D-CRT central axis, was 2.0 ± 1.0 mm (range 1.0–4.1 mm) for the included patients. A linear relationship between liver and chest wall motion amplitudes was observed for nine patients. One patient had a pronounced chest wall motion range compared to the liver motion amplitude (Fig. 4). The median respiratory periods ranged from 2.6 to 4.6 s, with 14 to 44 accepted respiratory cycles across the included patients. The average PTV volume was 1120 cc (range 627–1783 cc). The beam-on times of a single fraction were longer for 5-fraction plans. For example, the average beam-on times of 5 and 15-fraction RA plans were 136 s (range 121–148 s) and 83 s (range 77–93 s), respectively.

**Fig. 5:**
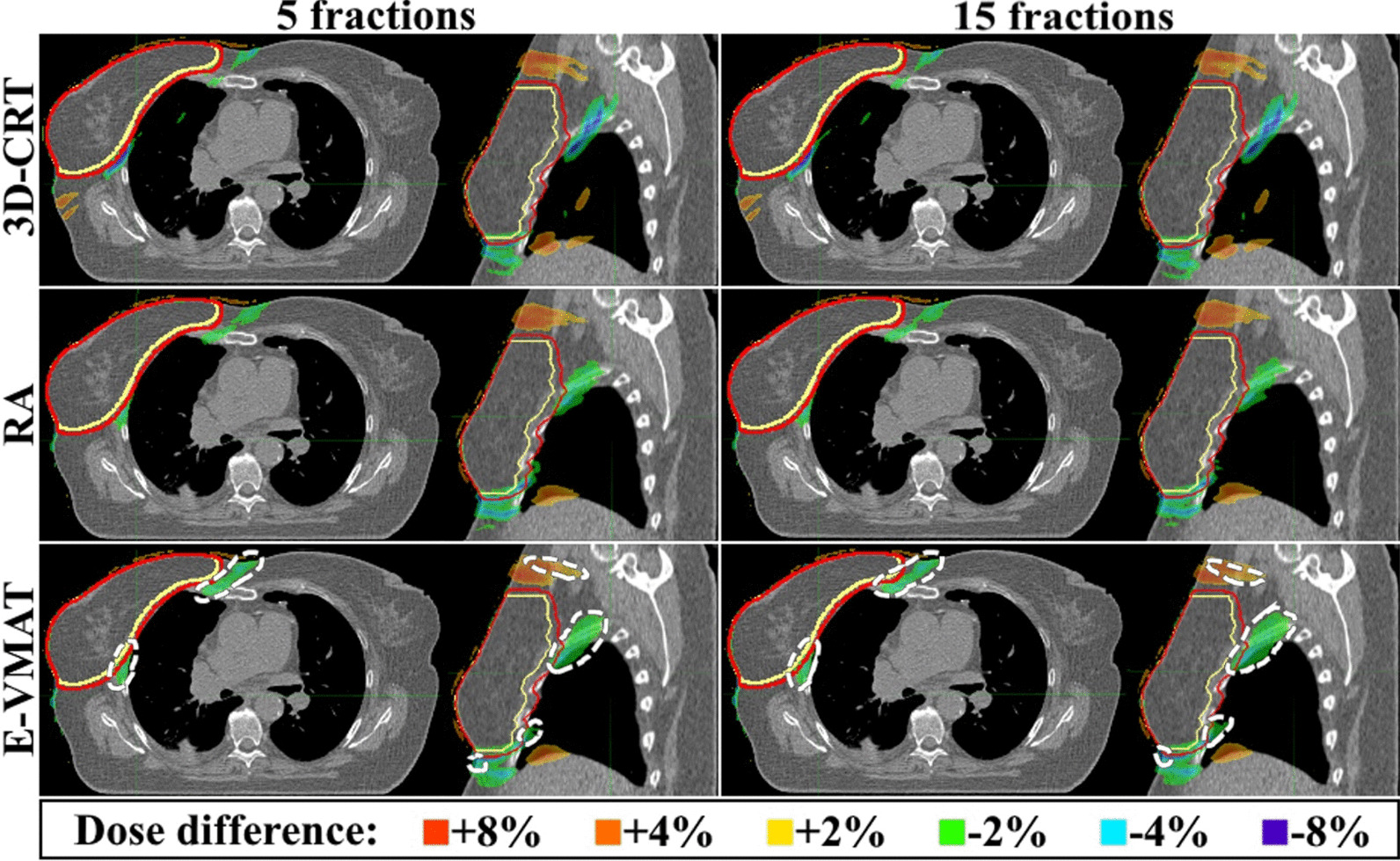
Axial and sagittal views of the average differential dose distribution deformed to one patient anatomy. The colored areas indicate the over-/underdose in the respiratory motion perturbed distribution compared to the planned as percentages of the prescribed dose. Statistically significant areas are encircled with dashed white lines (only for E-VMAT, *p* < 0.025). The clinical and planning target volume contours are illustrated in yellow and red, respectively

Respiratory motion induced a slight decrease in the PTVin coverage for RA (approximately 1.2%, p < 0.01) and E-VMAT (approximately 1.5%, *p* < 0.01), respectively (Table 1). Furthermore, a slight decrease in CTVin coverage was found for E-VMAT (approximately 0.4%, *p* < 0.01). The PTVin coverage was best retained by the 3D-CRT technique, for which the 5 and 15-fraction dose coverages decreased only by 0.2% (*p* = 0.30) and 0.3% (*p* = 0.31), respectively. The PTVin coverage was retained with RA in all included patients with both fractionations, whereas the coverage decreased below the planning goal in one 15-fraction case for 3D-CRT (from 96.0 to 94.8%) and 5 and 15-fraction cases for E-VMAT (96.5 to 94.4% and 96.5 to 94.5%) for one patient. CTVin coverage decreased below the planning goal only in the aforementioned 15-fraction 3D-CRT case (98.2 to 97.6%). Typically, the coverage decreased in the upper and lower medial parts of the PTV or lateral chest wall region (Fig. 5 and Additional files 1–6: Supplementary Animations 1–6).

**Table 1:** The dose-volume histogram parameters for the cropped planning and clinical target volumes (PTVin and CTVin)

		3D-CRT			RA			E-VMAT		
		Planned	Perturbed		Planned	Perturbed		Planned	Perturbed	
			5fr	15fr		5fr	15fr		5fr	15fr
PTVin	V95%	97.5 ± 1.5	97.3 ± 1.4	97.2 ± 1.4	98.0 ± 0.5	**96.9** ± 0.9*	**96.8** ± 0.9*	97.5 ± 0.9	**96.0** ± **1.1**	**96.0** ± **1.1**
	D1cc	108.0 ± 1.1	**107.5** ± **1.0**	**107.5** ± **1.1**	106.3 ± 0.9	**106.0 ± 1.0**	**106.0 ± 1.1**	104.6 ± 0.5	104.4 ± 0.5	**104.1 ± 0.8**
	Min1cc	91.9 ± 1.0	**90.8** ± **2.3**	**90.6** ± **2.3**	91.6 ± 1.0	**88.5** ± **3.8**	**88.4** ± **3.8**	85.0 ± 5.3	**80.6** ± **6.0**	**80.5** ± **6.0**
	CI	1.41 ± 0.14	**1.37** ± **0.14**	**1.37** ± **0.14**	1.16 ± 0.04	**1.12** ± 0.03*	**1.12** ± 0.03*	1.15 ± 0.07	**1.11** ± **0.06**	**1.10** ± **0.07**
	HI	10.9 ± 1.5	10.8 ± 1.5	10.8 ± 1.4	9.5 ± 0.6	**10.1** ± **0.8**	**10.1** ± **0.9**	8.6 ± 1.4	**10.4** ± **1.8**	**10.2** ± **1.8**
CTVin	V95%	98.8 ± 0.9	98.9 ± 0.6	98.9 ± 0.6	98.9 ± 0.5	98.9 ± 0.5	99.0 ± 0.5	99.0 ± 0.3	**98.6** ± **0.5**	**98.6** ± **0.5**
	D1cc	107.7 ± 1.2	107.4 ± 1.0	**107.3** ± **1.2**	106.2 ± 1.0	105.9 ± 1.0	105.9 ± 1.1	104.3 ± 0.4	104.3 ± 0.5	103.9 ± 0.7
	Min1cc	92.8 ± 1.1	92.7 ± 0.9	**92.6** ± **1.0**	92.6 ± 1.4	92.6 ± 1.5	92.5 ± 1.5	92.3 ± 1.1	**91.6 ± 0.9**	**91.4 ± 0.9**
	CI	1.75 ± 0.25	**1.71** ± **0.24**	**1.70** ± **0.24**	1.43 ± 0.13	**1.39** ± 0.11*	**1.38** ± 0.11*	1.43 ± 0.17	**1.37** ± **0.15**	**1.36** ± **0.15**
	HI	9.9 ± 1.6	**9.6** ± **1.5**	**9.6** ± **1.5**	8.9 ± 0.6	**8.6** ± **0.6**	**8.6** ± **0.6**	6.8 ± 0.8	**7.5** ± **0.9**	**7.3** ± **0.7**

The Planned and Perturbed columns indicate the planned and respiratory motion perturbed parameters, respectively. The units of V95, D1cc and Min% are presented as percentages of the prescribed dose. Statistical significance between the planned and respiratory motion perturbed dose is represented by bolding (*p* < 0.05) and between fracitonations by an asterisk (*p* < 0.05)

The HI of PTVin increased with both VMAT techniques (p < 0.01) while no significant change was observed with 3D-CRT (Table 1). Furthermore, the HI of CTVin decreased slightly with 3D-CRT and RA (*p* < 0.05), but on the contrary, an increase was observed for E-VMAT (*p* < 0.05). The CI of PTVin and CTVin decreased by 0.05–0.07 (*p* < 0.01) for all techniques.

The maximum dose to 1 cc volume in PTVin decreased for 3D-CRT (108.0 to 107.5%, *p* < 0.01) and RA (106.3 to 106.0%, *p* < 0.05), while statistically significant decrease was found for E-VMAT in the 15-fraction case (104.6 to 104.1%, *p* < 0.05). A decrease in D1cc for the CTVin was observed for 15-fraction 3D-CRT (107.7 to 107.3%, *p* < 0.05). The minimum dose to the 1 cc volume (Min1cc) in PTVin decreased for all techniques (*p* < 0.05, Table 1).

The D1cc of the contralateral breast decreased for all techniques (*p* < 0.05, Table 2). The D1cc of the ipsilateral lung decreased for all techniques (*p* < 0.05) except for the 5-fraction 3D-CRT. Small increases in mean doses were observed for contralateral breast, contralateral lung and heart for E-VMAT (*p* < 0.05). The mean dose for the contralateral breast decreased slightly for 3D-CRT and RA. Large variation in liver maximum dose was observed.

**Table 2:** Dose-volume histogram parameters of organs-at-risk

		3D-CRT			RA			E-VMAT		
		Planned	Perturbed		Plan	Perturbed		Planned	Perturbed	
			5fr	15fr		5fr	15fr		5fr	15fr
Lung R	V40%	12.6 ± 4.0	12.7 ± 4.2	12.7 ± 4.2	12.2 ± 1.3	12.1 ± . 1.5	12.1 ± 1.5	10.5 ± 2.6	10.4 ± 2.7	10.3 ± 2.7
	Mean	13.8 ± 3.6	13.8 ± 3.7	13.8 ± 3.7	14.1 ± 1.0	14.0 ± 1.2	14.0 ± 1.2	13.1 ± 2.0	13.2 ± 2.2	13.2 ± 2.2
	D1cc	97.4 ± 2.6	96.8 ± 2.5	**96.7** ± **2.6**	93.9 ± 2.9	**93.1** ± **3.0**	**93.1** ± **3.4**	92.9 ± 4.1	**91.9** ± **4.6**	**91.7** ± **4.5**
Lung L	D1cc	1.7 ± 0.7	1.7 ± 0.7	1.7 ± 0.7	3.6 ± 2.1	**3.5** ± **2.2**	**3.6** ± **2.2**	4.1 ± 2.1	4.2 ± 2.0	4.1 ± 2.0
	Mean	0.4 ± 0.1	0.4 ± 0.1	0.4 ± 0.1	0.5 ± 0.1	0.5 ± 0.1*	0.5 ± 0.1*	1.0 ± 0.3	**1.2** ± **0.3**	**1.2** ± **0.3**
Heart	D1cc	7.6 ± 8.0	7.7 ± 7.7	7.7 ± 7.7	10.2 ± 6.2	10.5 ± 6.3	10.5 ± 6.4	7.7 ± 4.9	7.8 ± 4.2	7.8 ± 4.2
	Mean	1.2 ± 0.2	1.2 ± 0.2	1.2 ± 0.2	1.6 ± 0.3	1.6 ± 0.3	1.6 ± 0.3	2.0 ± 0.4	**2.2** ± **0.4**	**2.2** ± **0.4**
Breast L	D1cc	5.7 ± 4.3	**5.1** ± **3.3**	**5.1** ± **3.3**	27.7 ± 11.1	**25.4** ± **9.3**	**25.4** ± **9.3**	12.7 ± 4.3	**11.6** ± **3.4**	**11.6** ± **3.4**
	Mean	0.6 ± 0.2	**0.6** ± **0.2**	**0.6** ± **0.2**	2.1 ± 0.7	**2.0** ± **0.6**	**2.0** ± **0.6**	1.6 ± 0.4	**1.7** ± **0.4**	**1.7** ± **0.4**
Liver	D1cc	31.5 ± 32.7	36.0 ± 34.4	35.9 ± 34.4	25.1 ± 27.9	27.7 ± 26.2	27.7 ± 26.1	22.7 ± 26.6	25.4 ± 24.8	25.4 ± 24.8
Body	D1cc	108.5 ± 1.1	**107.9** ± **1.0**	**107.8** ± **1.2**	106.5 ± 0.9	**106.1** ± **1.0**	**106.1 ± 1.1**	104.8 ± 0.6	104.5 ± 0.5*	**104.2** ± 0.8*

The Planned and Perturbed columns indicate the planned and respiratory motion perturbed parameters, respectively. The units of D1cc and Mean are presented as percentages of the prescribed dose. Statistical significance between the planned and respiratory motion perturbed dose is represented by bolding (*p* < 0.05) and between fractionations by an asterisk (*p* < 0.05)

Areas of underdose were observed inferior to the PTV, in the anterior and lateral chest wall region and between the PTV and sternum (Fig. 5). Slight overdose was observed superior to the PTV, in the superior part of liver and in the middle lobe of the ipsilateral lung. However, statistically significant areas were found only for E-VMAT (*p* < 0.025).

Statistical significance between the fractionations was found in PTVin (V95% and CI), CTVin (CI) and contralateral lung (mean) for RA and in Body for E-VMAT. However, the differences in DVH parameters between fractionations were small and estimated clinically irrelevant.

The PTVin coverage, HI and CI decreased the most for the patient that was excluded for all techniques (Additional file 7: Table S3). However, the CTVin coverage objective was retained as only small decreases were observed. The changes in PTVin and CTVin HI were the largest for RA and E-VMAT compared to other patients. The results regarding the OAR DVH parameters varied across the techniques (Additional file 7: Table S4). However, the largest increase in ipsilateral lung D1cc was observed for all techniques in this patient.

## Discussion

There were only slight differences between the static and respiratory motion perturbed VMAT distributions. Slight decreases in PTV dose coverage and homogeneity were observed for arc techniques, but the CTV dose coverage was retained on average. No significant decreases were found for PTV or CTV coverage when using 3D-CRT technique, which is in agreement with the previous studies utilizing tangential techniques [12–14].

The PTV dose coverage was mainly compromised in areas with steep dose fall-off gradients, such as the medial PTV region. The E-VMAT plans had a steep dose gradient in the medial part of PTV due to avoiding the contralateral breast and thus the most notable dose decrease was observed in the medial region of the PTV. The decrease might be avoided by decreasing the dose gradient in this region, although this might result in increased dose to contralateral tissue. The RA planning did not result in a similar steep dose gradient in this region and the dose decrease in the medial parts of CTV was thus smaller in volume. However, dose to the contralateral breast was higher compared to E-VMAT. Statistical significance was not found in the 3D-CRT or RA differential distributions (Fig. 5), mainly due to small patient cohort.

The CTVin coverage was also retained for the excluded patient, despite having the largest respiratory amplitude (5.3 mm) and period (6.1 s). However, the decrease in CTVin coverage was greater than on average for E-VMAT. However, the largest decrease in PTV dose coverage was found in this patient regardless of the technique (~ 2% for 3D-CRT, 4.1–4.7% for RA and E-VMAT). The respiratory amplitude of over 5 mm has been considered to have clinically significant impact in a study using the wedge technique [13].

Changes in HI of PTV indicated decreased homogeneity for VMAT plans. The CTVin dose homogeneity was conserved for 3D-CRT and RA techniques and only a small increase was observed for E-VMAT, even though the formula for HI is susceptible to changes in D2% and D98%. This suggests that respiratory motion increased dose heterogeneity in the areas of PTV edges, similar to observations for IMRT [17]. The PTV and CTV conformity increased consistently with respiratory motion for all patients.

The most significant changes in OAR parameters were observed for maximum doses (D1cc) as the changes in mean dose were marginal. Dose to the ipsilateral lung decreased slightly in the chest wall region and increased slightly in the center of the lower lobe. Furthermore, large variation in liver maximum dose was observed. This was expected since the liver might move into the fields with expiration.

On average, the DVH parameters determined for 5-fraction plans were equal to those determined for 15-fraction plans. For one patient, the perturbed 5-fraction 3D-CRT distribution retained the PTVin coverage goal while the corresponding 15-fraction distribution did not (difference of 0.7 percentage points (pp)). Similar effect was observed in the CTVin coverage of the same patient. In this study, ultra-hypofractionation resulted in longer beam-on times (136 s vs. 83 s, for RA), and the MLC patterns were identical as a function of gantry angle, because the 5-fraction plans were scaled from the 15-fraction plans with dose rate limit of 600 MU/min. The scaling approach was chosen as the scope of this study was to compare identical percentage dose distributions.

Some limitations exist in the study. Conducting the treatment planning on the end-inspiration image creates limitation, since free-breathing CT image is used for planning when respiratory gating is not used. However, variance in patient position was observed between the free-breathing image and 4D series in this patient cohort. The scope of this study was rather to simulate a worst-case scenario and thus end-inspiration image was used for planning. In addition, the included patients were initially treated for lung cancer and retrospectively selected for this study to investigate the effects of respiratory motion on the planned WBI dose distributions. However, as the patients' average chest wall motion range was similar to the breast cancer patients' respiratory motion range observed in previous studies [12, 13], the present patient group was considered suitable for the study. Furthermore, non-rigid fusions were used to transform and superimpose respiratory phase specific dose distributions to the end-inspiration phase. Inaccuracies in non-rigid fusions may generate uncertainty to simulated dose distributions and thus, exaggerate differences between planned and simulated distributions. To evaluate the quality of non-rigid fusion, all structures delineated to end-inspiration CT were deformed and transformed to other respiratory phases and quality of deformed structures was visually reviewed. Finally, it should be noted that other sources of error, such as tissue deformation [22], setup error [23] and variability in respiratory patterns [24, 25], were not considered in this study.

The aim of this study was to evaluate the dosimetric effect of respiratory motion in free-breathing WBI for VMAT techniques. While the PTV coverage and dose homogeneity declined, they were retained in the CTV in this worst-case scenario approach, when the respiratory motion amplitude of the chest wall is less than 5 mm. The general conclusion is that the homogeneous CTV dose is retained with moderate and ultra-hypofractionation, even though the dose is delivered from multiple angles. While other sources of error are present in a realistic breast cancer treatment, forming the PTV as a 5 mm expansion of CTV was sufficient in terms of respiratory motion induced error.

## Conclusion

Respiratory motion of less than 5 mm in magnitude did not result in clinically significant changes in the planned free-breathing WBI dose. The 5 mm margins were sufficient to account for the respiratory motion in terms of CTV dose homogeneity and coverage for VMAT. Steep dose gradients near the PTV edges might affect the CTV coverage. The 15 and 5-fraction approaches provided roughly the same dose-averaging results, as the increased fraction dose was combined with slower MLC speeds and longer beam-on times in this study.

## Supplementary Information

**Additional file 1**. Axial view animation of the deformed sum 5 fraction 3D-CRT differential distributions. The differential distributions of the 9 included patients were deformed to one patient's anatomy.

**Additional file 2**. Axial view animation of the deformed sum 15 fraction 3D-CRT differential distributions. The differential distributions of the 9 included patients were deformed to one patient's anatomy.

**Additional file 3**. Axial view animation of the deformed sum 5 fraction RA differential distributions. The differential distributions of the 9 included patients were deformed to one patient's anatomy.

**Additional file 4**. Axial view animation of the deformed sum 15 fraction RA differential distributions. The differential distributions of the 9 included patients were deformed to one patient's anatomy.

**Additional file 5**. Axial view animation of the deformed sum 5 fraction E-VMAT differential distributions. The differential distributions of the 9 included patients were deformed to one patient's anatomy.

**Additional file 6**. Axial view animation of the deformed sum 15 fraction E-VMAT differential distributions. The differential distributions of the 9 included patients were deformed to one patient's anatomy.

**Additional file 7**. Planned and respiratory motion perturbed dose parameters for the excluded patient.

## Data Availability

The datasets generated and analyzed during the current study are not publicly available due to protection of individual patient privacy but are available from the corresponding author on reasonable request and with permission of Central Finland Health Care District.

## Abbreviations

3D-CRT: Three-dimensional conformal radiation therapy
4D-CT: Four-dimensional computed tomography
AAA: Analytical anisotropic algorithm
CBCT: Cone-beam computed tomography
CI: Conformity index
CP: Control point
CTV: Clinical target volume
DVH: Dose-volume histogram
E-VMAT: Elekta VMAT
FB: Free-breathing
HI: Homogeneity index
IMRT: Intensity-modulated radiation therapy
MLC: Multi-leaf collimator
MU: Monitor unit
OAR: Organ-at-risk
PTV: Planning target volume
RA: Rapid arc
RPM: Real-time position management
TPS: Treatment planning system
VMAT: Volumetric modulated arc therapy
WBI: Whole-breast irradiation
