# Supplementary figures and images for: Dosimetric effect of respiratory motion on planned dose in whole-breast volumetric modulated arc therapy using moderate and ultra-hypofractionation
Source: Radiat Oncol. 2022 Mar 5;17:46. doi: 10.1186/s13014-022-02014-5 (PMC8898500; doi:10.1186/s13014-022-02014-5)

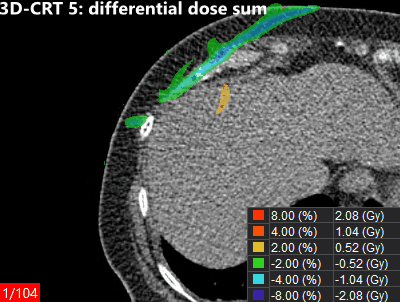

Supplement: Supplementary file 1 — Additional file 1. Axial view animation of the deformed sum 5 fraction 3D-CRT differential distributions. The differential distributions of the 9 included patients were deformed to one patient's anatomy. [file 13014_2022_2014_MOESM1_ESM.gif]

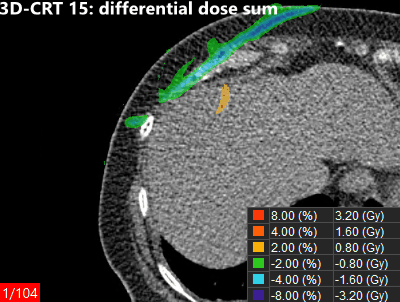

Supplement: Supplementary file 2 — Additional file 2. Axial view animation of the deformed sum 15 fraction 3D-CRT differential distributions. The differential distributions of the 9 included patients were deformed to one patient's anatomy. [file 13014_2022_2014_MOESM2_ESM.gif]

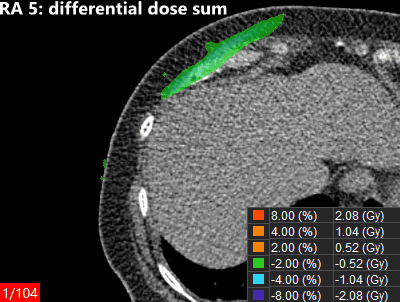

Supplement: Supplementary file 3 — Additional file 3. Axial view animation of the deformed sum 5 fraction RA differential distributions. The differential distributions of the 9 included patients were deformed to one patient's anatomy. [file 13014_2022_2014_MOESM3_ESM.gif]

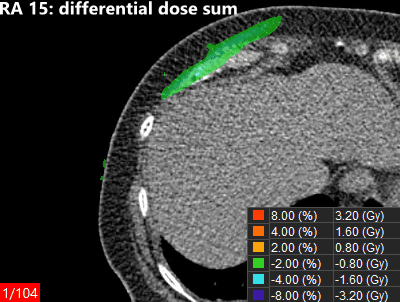

Supplement: Supplementary file 4 — Additional file 4. Axial view animation of the deformed sum 15 fraction RA differential distributions. The differential distributions of the 9 included patients were deformed to one patient's anatomy. [file 13014_2022_2014_MOESM4_ESM.gif]

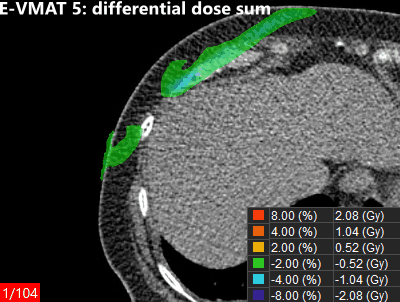

Supplement: Supplementary file 5 — Additional file 5. Axial view animation of the deformed sum 5 fraction E-VMAT differential distributions. The differential distributions of the 9 included patients were deformed to one patient's anatomy. [file 13014_2022_2014_MOESM5_ESM.gif]

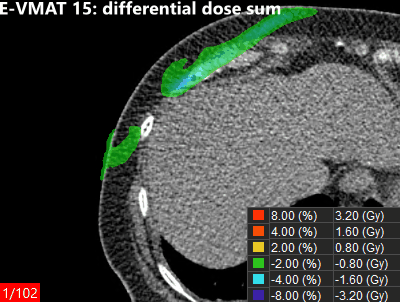

Supplement: Supplementary file 6 — Additional file 6. Axial view animation of the deformed sum 15 fraction E-VMAT differential distributions. The differential distributions of the 9 included patients were deformed to one patient's anatomy. [file 13014_2022_2014_MOESM6_ESM.gif]
