# Supplementary material for: Dosimetric effect of respiratory motion on planned dose in whole-breast volumetric modulated arc therapy using moderate and ultra-hypofractionation
Source: Radiat Oncol. 2022 Mar 5;17:46. doi: 10.1186/s13014-022-02014-5 (PMC8898500; doi:10.1186/s13014-022-02014-5)
Supplement: Supplementary file 7 — Additional file 7. Planned and respiratory motion perturbed dose parameters for the excluded patient. [file 13014_2022_2014_MOESM7_ESM.docx]

**Table 3:** The dose-volume parameters for the cropped planning and clinical target volume (PTVin and CTVin) for the excluded patient

|  |  | 3D-CRT | | | RA | | | E-VMAT | | |
| --- | --- | --- | --- | --- | --- | --- | --- | --- | --- | --- |
|  |  | Planned | Perturbed | | Planned | Perturbed | | Planned | Perturbed | |
|  |  |  | 5fr | 15fr |  | 5fr | 15fr |  | 5fr | 15fr |
| PTVin | V95% | 98.0 | 95.9 | 96.1 | 98.5 | 93.9 | 94.1 | 98.4 | 94.4 | 94.2 |
|  | D1cc | 108.8 | 107.6 | 107.7 | 105.7 | 106.4 | 106.4 | 103.9 | 104.5 | 104.5 |
|  | Min1cc | 92.7 | 84.8 | 84.6 | 91.2 | 81.2 | 81.6 | 89.3 | 76.6 | 76.2 |
|  | CI | 1.40 | 1.28 | 1.28 | 1.15 | 1.05 | 1.05 | 1.18 | 1.07 | 1.06 |
|  | HI | 11.4 | 11.2 | 11.2 | 8.7 | 12.5 | 12.6 | 7.3 | 12.4 | 12.5 |
| CTVin | V95% | 99.4 | 99.2 | 99.2 | 98.8 | 98.9 | 99.1 | 99.4 | 98.8 | 98.7 |
|  | D1cc | 108.5 | 107.4 | 107.4 | 105.5 | 106.4 | 106.7 | 103.9 | 104.5 | 104.5 |
|  | Min1cc | 93.6 | 93.6 | 93.7 | 91.4 | 90.7 | 91.2 | 93.3 | 92.1 | 91.9 |
|  | CI | 1.7 | 1.6 | 1.6 | 1.4 | 1.3 | 1.3 | 1.5 | 1.3 | 1.3 |
|  | HI | 10.0 | 9.1 | 9.1 | 8.3 | 8.6 | 8.5 | 6.2 | 7.7 | 7.8 |

Table 3 legend: The Planned and Perturbed columns indicate the planned and respiratory motion perturbed parameters, respectively. The units of V95, D1cc and Min1cc are presented as percentages of the prescribed dose. V95 is the volume percentage receiving at least 95% of the prescribed dose. D1cc is the maximum isodose that covers at least a volume of 1 cc of the present structure. Min1cc is the minimum isodose that covers at least 1 cc.

**Table 4:** The dose-volume parameters for the organs-at-risk for the excluded patient

|  |  | 3D-CRT | | | RA | | | E-VMAT | | |
| --- | --- | --- | --- | --- | --- | --- | --- | --- | --- | --- |
|  |  | Planned | Perturbed | | Plan | Perturbed | | Planned | Perturbed | |
|  |  |  | 5fr | 15fr |  | 5fr | 15fr |  | 5fr | 15fr |
| Lung R | V40% | 10.3 | 10.3 | 10.3 | 9.5 | 9.1 | 9.2 | 9.0 | 8.2 | 8.2 |
|  | Mean | 11.7 | 11.4 | 11.5 | 11.8 | 11.4 | 11.4 | 11.7 | 11.2 | 11.2 |
|  | D1cc | 96.4 | 94.4 | 94.5 | 91.8 | 88.1 | 88.0 | 90.0 | 85.6 | 85.6 |
| Lung L | D1cc | 2.1 | 1.9 | 1.9 | 5.1 | 4.2 | 4.3 | 3.7 | 3.6 | 3.6 |
|  | Mean | 0.4 | 0.4 | 0.4 | 0.7 | 0.6 | 0.6 | 1.0 | 1.2 | 1.2 |
| Heart | D1cc | 5.7 | 5.6 | 5.6 | 7.9 | 7.3 | 7.4 | 6.3 | 6.2 | 6.2 |
|  | Mean | 1.1 | 1.0 | 1.0 | 1.5 | 1.5 | 1.5 | 1.8 | 2.0 | 2.0 |
| Breast L | D1cc | 4.5 | 4.3 | 4.2 | 22.1 | 20.5 | 20.5 | 9.1 | 8.6 | 8.6 |
|  | Mean | 0.5 | 0.5 | 0.5 | 1.9 | 1.7 | 1.7 | 1.4 | 1.4 | 1.4 |
| Liver | D1cc | 9.0 | 25.1 | 25.6 | 16.1 | 32.2 | 32.1 | 14.5 | 32.8 | 32.8 |
| Body | D1cc | 109.4 | 108.0 | 108.1 | 105.9 | 106.5 | 106..8 | 104.1 | 104.5 | 104.5 |

Table 4 legend: The Planned and Perturbed columns indicate the planned and respiratory motion perturbed parameters, respectively. The units of D1cc and Mean are presented as percentages of the prescribed dose. V40% is the isodose that covers 40% of the present structure volume. D1cc is the maximum isodose that covers at least a volume of 1 cc of the present structure.
